# Hidden Blood Loss and Its Risk Factors for Oblique Lumbar Interbody Fusion

**DOI:** 10.3390/jcm13051454

**Published:** 2024-03-02

**Authors:** Jae Hyuk Yang, Hong Jin Kim, Minsu An, Seung Woo Suh, Dong Gune Chang

**Affiliations:** 1 Department of Orthopaedic Surgery, Korea University Anam Hospital, College of Medicine, Korea University, Seoul 02841, Republic of Korea; kuspine@korea.ac.kr (J.H.Y.); toavakama@naver.com (M.A.); 2 Department of Orthopaedic Surgery, Inje University Sanggye Paik Hospital, College of Medicine, Inje University, Seoul 01757, Republic of Korea; hongjin0925@naver.com; 3 Department of Orthopedic Surgery, Korea University Guro Hospital, College of Medicine, Korea University, Seoul 08308, Republic of Korea; spine@korea.ac.kr

**Keywords:** hidden blood loss, hemoglobin loss, oblique lumbar interbody fusion, pedicle screw fixation type, degenerative lumbar disease

## Abstract

(1) **Background:** The amount of blood loss during oblique lumber interbody fusion (OLIF) surgery is often underestimated and may contribute to adverse postoperative outcomes. This study aims to evaluate hidden blood loss (HBL) in patients who underwent OLIF for degenerative lumbar spine disease and to analyze its risk factors. (2) **Methods:** The medical records of 179 patients who underwent OLIF surgery from 2015 to 2022 were reviewed. The HBL and total blood loss (TBL) were estimated using the Gross formula. Pearson correlation, Spearman correlation, and multivariate linear regression analyses were used to investigate risk factors for HBL. (3) **Results:** The mean HBL was 675.2 mL, and the mean hemoglobin loss was 1.7 g/dL during OLIF surgery. In the multivariate linear regression analysis, TBL (*p* < 0.001), estimated blood loss (*p* < 0.001), and pedicle screw fixation type (*p* = 0.039) were identified as independent risk factors of HBL. (4) **Conclusions:** The OLIF is associated with substantial perioperative HBL, for which we identified risk factors of TBL, EBL, and pedicle screw fixation type. Notably, OLIF with percutaneous pedicle screw fixation resulted in greater HBL than stand-alone OLIF or OLIF with open pedicle screw fixation.

## 1. Introduction

Substantial blood loss during spinal surgery can lead to various complications, including postoperative anemia, prolonged hospital stays, and increased morbidity and mortality [1,2,3,4,5]. Surgical management of degenerative spine disease using interbody fusion has increased significantly, but the traditional posterior approach is associated with a large amount of blood loss and requires massive blood transfusion [1,6]. For these reasons, various surgical approaches including minimally invasive spinal surgery have been introduced to reduce intraoperative bleeding and to prevent postoperative complications [7,8,9,10,11,12].

Recently, oblique lumbar interbody fusion (OLIF) has gained popularity as a spinal arthrodesis technique for the treatment of degenerative lumbar disease [6]. The efficacy and safety of OLIF procedures have been demonstrated in many previous studies, particularly with respect to the reduction in intraoperative bleeding loss and invasive soft tissue injury, which also contribute to more rapid recovery after surgery [2,6,8]. However, undetectable blood loss, also called hidden blood loss (HBL), during OLIF procedures is often neglected by spine surgeons, resulting in unexpected postoperative complications [5,6,13,14,15,16,17,18,19,20].

The major causes of HBL during OLIF are the extravasation of blood into interstitial tissue and blood loss from hemolysis in the retroperitoneal space, the volumes of which are not included in estimates of intraoperative blood loss (EBL) or postoperative drainage [6,20]. It is essential to understand all sources of blood loss in OLIF and to accurately measure HBL to prevent unexpected post-OLIF complications. Therefore, we conducted a multi-center retrospective case series study of patients who underwent OLIF surgery, evaluated HBL during the procedure, and identified risk factors for HBL during OLIF procedures.

## 2. Materials and Methods

This study was a retrospective analysis conducted at two centers, and the concept and procedures for this study were approved by the Institutional Review Board (IRB number: K2024-0116-001). This retrospective study was also conducted by the Strengthening the Reporting of Observational Studies in Epidemiology (STROBE) guidelines [21].

All patients underwent OLIF surgeries performed by two senior surgeons with extensive experience in the procedure. The operative indications of OLIF in this study were not different from that of LIF procedures with the extension trend of surgical indication. Thus, all patients with degenerative lumbar spinal disorders (severe spinal stenosis, herniated intervertebral disc, and/or spondylolisthesis) were subjected to surgical treatment. Therefore, the operative indication in this study was the patients with degenerative lumbar spinal disorders who had no response to conservative treatment for more than 3 months or had any neurological deficits. For surgical procedures of OLIF, with a right lateral decubitus position after general anesthesia, about 4 cm skin incision centered on the marked disc level was made, parallel to the external oblique muscle fibers under the C-arm fluoroscopic guidance. The blunt soft tissue dissection of the external oblique, internal oblique, and transverse abdominal muscles allowed access to the retroperitoneal space. The psoas muscle was identified and retracted posteriorly to expose the operation window for intervertebral disc space. Afterward, a Kirschner wire was placed into the target and confirmed under the C-arm fluoroscopic guidance. The tubular retractor was inserted and docked after applying serial dilators. The intervertebral disc space and adjacent vertebral bodies were exposed after docking the tubular retractor. Then, the disc and cartilaginous endplates were removed. A cage filled with synthetic bone graft substitutes was inserted into the intervertebral disc space under the C-arm fluoroscopic guidance. In the case of percutaneous pedicle screw fixation, the patient was turned to the prone position, and the tip of the puncture needle was initially placed on the outer edge of the projection of the pedicle of the vertebral arch (10 o’clock on the left and 2 o’clock on the right) after blunt dissection of the space between the longissimus and multifidus muscle. The guidewire, expanding tube, and protective sleeve were placed, step by step. All pedicle screws were inserted into the vertebral body through the guidewire under the C-arm fluoroscopic guidance. After pedicle screw fixation, the pre-bended rod was connected to the pedicle screw by tightening the nuts.

**Figure 1:**
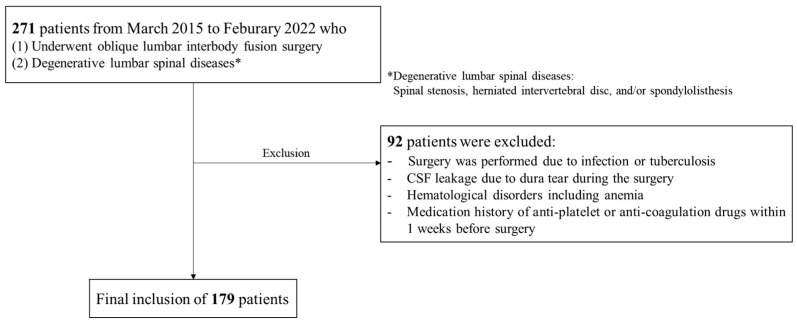
Flowchart of this study.

The medical records of 271 patients who underwent OLIF surgery for degenerative lumbar spinal conditions, including spinal stenosis, herniated intervertebral disc, and/or spondylolisthesis, were collected from March 2015 to February 2022. The exclusion criteria for this study were as follows: (1) surgery was performed due to infection or tuberculosis, (2) CSF leakage due to dura tear during the surgery, (3) history of hematologic disorder including anemia, and (4) medication history of anti-platelet or anti-coagulation drugs within one week before surgery. A total of 179 patients were finally included, and data from those patients were analyzed (Figure 1).

All patient data were collected from the hospital database, and a retrospective analysis was performed. The captured demographic and operative variables were sex, age, height, weight, body mass index (BMI), underlying disease (hypertension, diabetic mellitus), disease type (spinal stenosis, spondylolisthesis, or mixed), American Society of Anesthesiologist classification, operative time, interbody fusion level (one-level, two-level, or multi-level), interbody fusion type (OLIF only or OLIF combined [OLIF with other interbody fusion procedures]), pedicle screw fixation type (no pedicle screw fixation [stand-alone], percutaneous pedicle screw fixation [PPF], open pedicle screw fixation), and perioperative complications including endplate breakage and/or anterior longitudinal ligament (ALL) rupture. The laboratory data included hemoglobin (Hb) concentration, hematocrit (Hct), platelet count, prothrombin time (PT), PT international normalized ratio (INR), activated partial thromboplastin time (aPTT), and amount of drainage.

To evaluate blood loss, the patient’s blood volume (PBV) was calculated using the formula from Nadler et al.: PBV (L) = (k_1_ × height(m) ^3^) + (k_2_ × weight(kg)) + k_3_, where k_1_ = 0.3669, k_2_ = 0.03219, and k_3_ = 0.6041 for males, and k_1_ = 0.3561, k_2_ = 0.03308, and k_3_ = 0.1833 for females [22]. Total blood loss (TBL) was calculated using the Gross formula: TBL (L) = PBV (L) × (preoperative Hct − postoperative Hct)/average Hct [23]. The postoperative Hct was defined as the lowest Hct value measured between postoperative days two and four. The average Hct was defined as the average of the preoperative and postoperative Hct values. Postoperative Hb loss (Hb_loss_) was calculated using the following formula: Hbloss (g/dL) = preoperative Hb − postoperative Hb. The EBL was calculated by summing blood draining into the suction bottle and that collected from surgical gauze. Finally, HBL was calculated as follows: HBL (L) = (TBL (L) + blood transfusion volume (L)) − (measured bone loss including EBL + amount of drainage).

All statistical analyses were performed using SPSS^®^ Statistics for Windows, version 21.0 (IBM Corp., Armonk, NY, USA). The normality of the data distribution was confirmed by the Kolmogorov–Smirnov test. All parametric values are expressed as mean\pm standard deviation, and all non-parametric values are expressed as number (percentage). After confirming data homogeneity or heteroscedasticity, Student’s *t*-test was used for continuous variables to compare two subgroups. Comparisons of three groups were performed using a one-way repeated measures analysis of variance, and the post hoc analysis used the Bonferroni test. Pearson’s correlation or Spearman’s correlation analyses were performed to confirm correlations between HBL and the identified risk factors. Multivariate linear regression analysis was performed to assess the risk factors for HBL. To perform the multivariate linear regression analysis, we initially performed the univariate regression linear analysis for significant variables in the result of the correlation analysis. Then, we selected the statistically significant variables from the univariate regression linear analysis, which were used for the multivariate regression analysis. Statistical significance was set at *p* < 0.05.

## 3. Results

### 3.1. Baseline Characteristics

All demographic and operative data for the study patients are summarized in Table 1. The mean age was 67.8 years, and the mean BMI was 25.0 kg/m^2^. Regarding the underlying disease, 52.5% of the patients had hypertension, and 24.6% had diabetes mellitus. Regarding the spinal disease type, 70.4% of the patients had spinal stenosis, 14.5% had spondylolisthesis, and 13.4% had spinal stenosis and spondylolisthesis. The mean operative time was 237.1 min. One-, two-, or multi-level OLIF interbody fusion was performed in 52.5%, 28.5%, and 19.0% of the patients, respectively. Most patients (87.2%) underwent OLIF only, with the remaining 12.8% undergoing OLIF with anterior lumbar interbody fusion (ALIF), transforaminal lumbar interbody fusion (TLIF), or posterior lumber interbody fusion (PLIF). Moreover, 17.9% of the total patients (32 of 179) received intraoperative blood transfusions. Of these, 50.0%, 5.0%, and 37.5% of patients who underwent stand-alone OLIF (17 of 34), PPF (6 of 121), and open pedicle screw fixation (9 of 24) received intraoperative transfusion, respectively. Regarding the pedicle screw fixation type, 19.0%, 67.6%, and 13.4% of all patients underwent stand-alone OLIF, OLIF with PPF, or OLIF with open pedicle screw fixation and blood transfusion, respectively (Table 1).

### 3.2. Blood Loss

**Figure 2:**
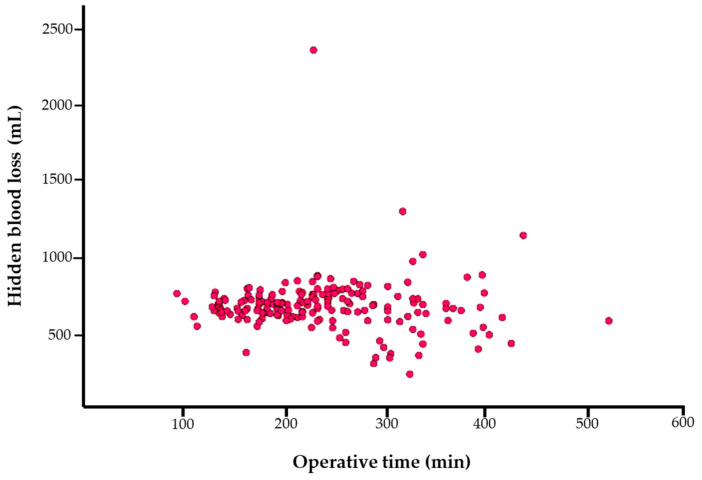
Scatter plot of operative time versus hidden blood loss during oblique lumbar interbody fusion procedures.

In this study, the estimated HBL was 675.2 mL and Hb_loss_ was 1.7 g/dL (Table 2). There was no tendency for HBL to increase as the operative time for OLIF procedures increased (Figure 2).

**Figure 3:**
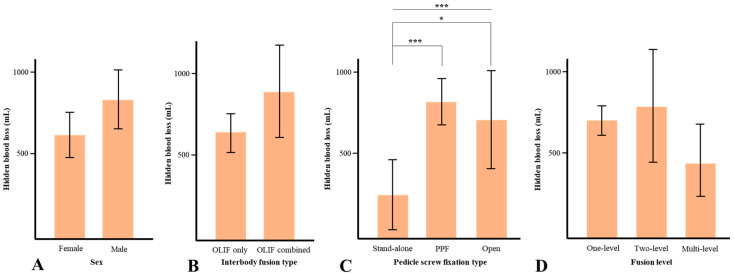
Hidden blood loss according to sex (**A**), interbody fusion type (**B**), pedicle screw fixation type (**C**), and fusion level (**D**). * *p* < 0.05, and *** *p* < 0.005.

No significant differences in HBL were found according to sex (*p* = 0.079), interbody fusion type (*p* = 0.187), and fusion level (*p* = 0.097). For the pedicle screw fixation type, HBL was 252.5 mL for stand-alone OLIF, 790 mL for OLIF with PPF, and 695.2 mL for OLIF with open pedicle screw fixation (*p* = 0.001). The Bonferroni post hoc analysis for the pedicle screw fixation type showed statistical differences except between OLIF with PPF and OLIF with open pedicle screw fixation (Figure 3). 

**Figure 4:**
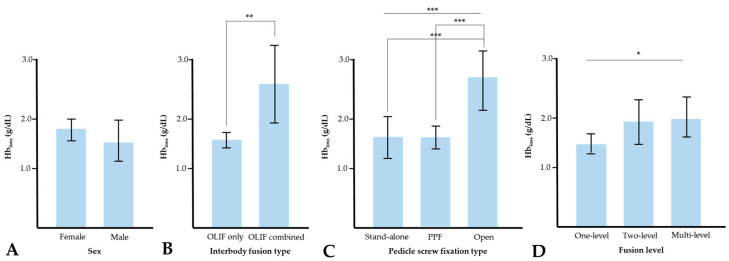
Hemoglobin loss according to sex (**A**), interbody fusion type (**B**), pedicle screw fixation type (**C**), and fusion level (**D**). * *p* < 0.05, ** *p* < 0.01, and *** *p* < 0.005. Hb_loss_ = hemoglobin loss.

There was no significant difference in Hb_loss_ according to the sex of the patients (*p* = 0.278). However, Hb_loss_ was significantly greater (*p* = 0.008) in patients who underwent only an OLIF procedure (2.6 g/dL) than in those who underwent OLIF together with other interbody fusion procedures (1.6 g/dL). In addition, Hb_loss_ was significantly greater (*p* < 0.001) in patients who underwent OLIF with open pedicle screw fixation (2.7 g/dL) than in those who underwent stand-alone OLIF (1.6 g/dL) or OLIF with PPF (1.6 g/dL). Bonferroni post hoc analyses showed statistical differences except between stand-alone OLIF and OLIF with PPF. As the fusion level increased, Hb_loss_ also increased significantly (*p* = 0.031), but Bonferroni post hoc analysis found no significant difference (Figure 4). The perioperative laboratory data are presented in Table 3.

### 3.3. Correlation Analysis and Multivariate Linear Regression Analysis for HBL

The results of the correlation analysis for HBL are presented in Table 4. Among the factors significantly associated with HBL, TBL had the greatest positive correlation (r = 0.876, *p* < 0.001), while EBL was moderately negatively correlated with HBL (r = −0.447, *p* < 0.001) in Pearson’s correlation analysis. The pedicle screw fixation type showed a significant, though small, positive correlation with HBL (r = 0.214, *p* < 0.001). To better identify the most important independent risk factors for HBL during OLIF among those identified by the correlation analysis in Table 4, a multi-linear regression analysis was performed and showed statistical significance for three factors, as shown in Table 5: TBL (Beta = 0.996, *p* < 0.001), EBL (Beta = −0.935, *p* < 0.001), and pedicle screw fixation type (Beta = 11.256, *p* = 0.039).

## 4. Discussion

Surgical techniques to treat complex spinal diseases have advanced dramatically in the past two decades [2]. However, perioperative blood loss in spine surgery remains a major concern because a massive transfusion is often required to address it, and blood loss increases infection risk, morbidity, and mortality [1]. Since the OLIF procedure was first introduced by Silvestre in 2012, it has become established as a minimally invasive spinal fusion technique to treat degenerative diseases of the lumbar spine [6]. Many OLIF surgical options, such as OLIF with PPF and OLIF with other interbody fusion procedures, are widely used [6]. However, these OLIF-based techniques can easily mask blood loss [20,24]. Therefore, we studied HBL in a study population of 179 patients who underwent OLIF procedures in a multi-center retrospective study and identified risk factors for HBL during the procedure. In our study, the mean HBL was 675.2 mL, which was higher than the volume (558 mL) reported by Zhu et al., although they investigated only one-level OLIF [20]. 

The advent of minimally invasive spinal surgery employing an anterior approach contributed to the further development of lumbar spinal surgical techniques to reduce intraoperative blood loss and complications [1,2,14]. Our findings indicated a nearly constant amount of HBL during the OLIF procedures regardless of operative time. The surgical approach in the OLIF procedure is to access the intervertebral space by way of the anatomical space between the inferior vena cava and the psoas muscle [6]. The advantage of the anterior approach is that it minimizes blood loss from adjacent bone and tissue injuries, which may explain the similar HBL volumes regardless of operative time. Furthermore, Wen et al. reported that, in PLIF procedures, HBL increased disproportionately as the operative time increased [25]. 

Our findings provide some insight regarding the relationships between HBL and the pedicle screw fixation type. Our results suggest that PPF may lead to large HBL volumes. There have been two previous reports of large volumes of HBL when treating thoracolumbar fractures using PPF. Since PPF-based procedures are performed using the Wiltse approach, there is a greater likelihood of muscle bleeding [7,24,26]. Furthermore, it is difficult to accurately measure visible blood loss during minimally invasive procedures, which was reflected as HBL rather than EBL in our study. In our study, hemoglobin loss did not differ significantly between patients who underwent stand-alone OLIF and those who underwent OLIF with PPF. A large HBL volume for OLIF with PPF procedures was not definitively detected, even in our laboratory findings, indicating the need for caution during perioperative patient management. Overall, our findings indicate that OLIF with PPF contributed to large HBL volumes, and special attention during the perioperative phase is essential for patients who undergo OLIF with PPF. 

Our study showed that HBL volumes did not differ significantly between the three fusion levels assessed (*p* = 0.097). Furthermore, the interbody fusion level was not a significant influencing factor for HBL (r = −0.101, *p* = 0.083). Therefore, HBL during OLIF procedures was not affected by the fusion level. This result is consistent with the characterization of OLIF as a minimally invasive spinal surgery, and even this technique is not approached as an intra-muscular plane to reduce adjacent muscle injuries [5,9]. Furthermore, the mean HBL volume for multi-level fusion cases (448.0 mL) was lower than that for one-level (703.6 mL) and two-level (789.2 mL) cases. Though this might seem unexpected, more patients who underwent multi-level OLIF also had open pedicle screw fixation, and we postulate the blood loss, in such cases, was reflected more accurately in the EBL volume than as HBL. Among patients who underwent one- or two-level OLIF, OLIF with PPF was more frequent than OLIF with open pedicle screw fixation as an independent risk factor of HBL.

Combined interbody fusion procedures (i.e., OLIF with other interbody fusion) did not contribute to increased HBL. Furthermore, the interbody fusion type was not correlated with HBL and was not an influencing factor in this study. Stand-alone OLIF showed lower HBL (252.5 mL), indicating that interbody fusion procedures do not result in higher HBL. Therefore, regardless of the type (e.g., ALIF, TLIF, or OLIF), interbody fusion is a safe procedure concerning the risk of bleeding.

The factors influencing HBL in OLIF in this study included some parameters regarding blood volume and laboratory findings. Of these influencing factors, TBL showed a strong correlation with HBL. The TBL volume was calculated based on the Gross formula, which includes factors of height, weight, and Hct, which were also found to be influencing factors for HBL in this study. The EBL itself is not typically large in OLIF procedures and is zero in most cases. This indicates a lower correlation with EBL (moderate) than with TBL (high). Furthermore, hemoglobin loss showed a low correlation with HBL (r = 0.386), indicating that hemoglobin loss may not reflect HBL well after OLIF surgery.

The independent risk factors for HBL in OLIR surgery were TBL, EBL, and pedicle screw fixation, indicating that PPF may potentially result in large HBL. One study suggested that the thickness of the abdominal wall soft tissue is an independent risk factor for HCL during one-level OLIF procedures [20]. That study explained that the abdominal muscle approach was prone not only to blood loss but also to greater permeability for blood extravasation [20]. Our study data did not include this radiological factor but suggested that PPF itself was associated with greater HBL because the procedure does not allow for the measurement of EBL and intraoperative bleeding into the surrounding tissue. Complex spinal diseases such as adult spinal deformity are increasing, and the demand for minimally invasive surgery such as OLIF increases [2]. Therefore, surgeons should keep in mind that there may be a large amount of undetectable bleeding during PPF procedures.

This study had some limitations. First, this study used a retrospective design. The retrospective design inherently limits the ability to establish causality between identified risk factors and HBL because it is associated with selection bias as the data may not be representative of all patients undergoing OLIF. Especially, prospective research for this subject could provide robust data by controlling confounding variables more effectively, which offers insight into the temporal relationship between OLIF surgery and HBL. Second, a small number of subgroups was identified in this study despite the large study population (power = 0.99 for the 179-patient sample size). Meanwhile, our data were based on the patients who underwent OLIF surgery in two centers, which may limit the applicability of the results to broader clinical settings and influence the generalizability of the results. However, the data, surgically performed by two senior surgeons in two centers and determined by a single healthcare system (Korean National Health Insurance Service), may minimize these limitations in our retrospective design. There also may be unmeasured variables that could affect the relationship between the identified risk factors and HBL from baseline health status to medication use and other comorbidities. Therefore, a large sample-sized prospective study will be required to verify our results in the future. Although our study has limitations regarding study design, considerable HBL after OLIF surgery can provide meaningful academical value as preliminary data before performing the new study using a prospective design. Third, it is possible that some laboratory results were underestimated due to hemodilution via intravenous fluid infusion during the perioperative period. Fourth, our analysis to evaluate HBL relies on formulas and indirect measurements, which may potentially not capture the exact extent of blood loss. The process of performing EBL measurements in the operating room could be prone to errors or inaccuracies. Therefore, objective criteria for the measurement of blood loss are needed to evaluate the exact HBL, particularly in the context of complex spinal surgeries [27]. Last, our study did not include radiological findings such as the assessment of abdominal thickness or paraspinal muscle quality, and a future trial is needed to investigate radiological risk factors for HBL during OLIF surgery.

## 5. Conclusions

The OLIF procedures were associated with substantial perioperative HBL, for which the risk factors of TBL, EBL, and pedicle screw fixation type were identified. Notably, OLIF with PPF was associated with greater HBL than it was for stand-alone OLIF or OLIF with open pedicle screw fixation. Therefore, the selection of the pedicle screw fixation type for OLIF surgery should be carefully considered, and patients should be monitored for blood loss during the perioperative period.

## Figures and Tables

**Table 1:** Baseline characteristics for the patients who underwent OLIF surgery.

Variables	Cases (*n* = 179)
Age (years)	67.8 ± 8.9 *
Sex (*n*) Female Male	129 (72.1%) 50 (27.9%)
Height (cm)	156.3 ± 9.0 *
Weight (kg)	61.1 ± 11.5 *
BMI (kg/m^2^)	25.0 ± 3.7 *
Underlying disease (*n*) Hypertension Diabetic mellitus	94 (52.5%) 44 (24.6%)
Disease type (*n*) Spinal stenosis Spondylolisthesis Mixed	129 (70.4%) 26 (14.5%) 24 (13.4%)
ASA classification (*n*) I II III	8 (4.5%) 124 (69.3%) 47 (26.2%)
Operative time (min)	237.1 ± 78.8 *
Interbody fusion level (*n*) One-level Two-level Multi-level	94 (52.5%) 51 (28.5%) 34 (19.0%)
Interbody fusion type (*n*) OLIF only OLIF combined (OLIF + ALIF/TLIF/PLIF)	158 (87.2%) 23 (12.8%)
Pedicle screw fixation type (*n*) Stand-alone PPF Open pedicle screw fixation	34 (19.0%) 121 (67.6%) 24 (13.4%)
Perioperative complications (*n*) Endplate breakage ALL rupture Blood transfusion (*n*)	38 (21.0%) 21 (11.7%) 32 (17.9%)

* Data = mean ± standard deviation. OLIF = oblique lumbar interbody fusion; *n* = number; BMI = body mass index; ASA = American Society of Anesthesiologist; ALIF = anterior lumbar interbody fusion; TLIF = transforaminal lumbar interbody fusion; PLIF = posterior lumbar interbody fusion; PPF = percutaneous pedicle screw fixation; ALL = anterior longitudinal ligament.

**Table 2:** The blood loss for the patients who underwent OLIF surgery.

Variables	Cases (*n* = 179)
EBL (mL)	179.3 ± 374.0
TBL (mL)	846.7 ± 663.8
HBL (mL)	675.2 ± 741.4
Drain amount (mL)	115.2 ± 175.1
Hb_loss_ (g/dL)	1.7 ± 1.1

All data = mean ± standard deviation. OLIF = oblique lumbar interbody fusion; *n* = number; EBL = estimated blood loss; TBL = total blood loss; HBL = hidden blood loss; Hb = hemoglobin.

**Table 3:** Perioperative blood loss and laboratory data for the patients who underwent OLIF surgery.

Variable	Preop Day	Op Day	POD 1	POD 2	POD 4	POD 7
Hb (g/dL)	13.2 ± 1.3	12.0 ± 1.3	11.5 ± 1.3	11.0 ± 1.4	10.7 ± 1.4	11.0 ± 1.5
Hct (%)	39.7 ± 3.8	36.1 ± 3.8	36.0 ± 23.9	32.6 ± 4.0	31.4 ± 4.9	32.9 ± 4.2
Platelet (×10^3^/µL)	243.7 ± 69.0	224.0 ± 132.4	196.5 ± 55.5	177.7 ± 49.6	214.1 ± 61.6	267.9 ± 77.8
PT (s)	12.7 ± 0.6	13.5 ± 0.7	15.4 ± 13.7	14.2 ± 1.6	13.6 ± 0.9	13.4 ± 0.8
PT INR	1.0 ± 0.1	1.0 ± 0.1	1.1 ± 0.1	1.5 ± 0.3	1.1 ± 0.1	1.0 ± 0.1
aPTT (s)	33.9 ± 5.9	32.8 ± 6.5	35.5 ± 5.3	42.0 ± 11.6	50.8 ± 14.6	47.4 ± 13.2
Drain amount (mL)	N/A	N/A	92.8 ± 149.1	42.8 ± 51.4	42.8 ± 51.4	N/A

All data = mean ± standard deviation. Preop = preoperative; Op = operative; POD = postoperative day; Hb = hemoglobin; Hct = hematocrit; PT = prothrombin time; INR = international normalized ratio; aPTT = activated partial thromboplastin time.

**Table 4:** Correlation analysis for HBL during OLIF surgery.

Variables	Correlation Coefficient	*p*
Age	−0.036	0.634
Height	0.234	0.002
Weight	0.163	0.030
BMI	0.015	0.845
Operative time	−0.058	0.441
Preoperative Hb	0.280	<0.001
Preoperative Hct	0.288	<0.001
Preoperative platelet	−0.108	0.152
PT	0.022	0.775
PT INR	0.046	0.541
aPTT	−0.043	0.568
TBL	0.876	<0.001
EBL	−0.447	<0.001
Hb_loss_	0.386	<0.001
Sex	0.171	0.005
Hypertension	−0.109	0.076
Diabetic mellitus	0.047	0.443
Disease type	−0.106	0.074
ASA classification	−0.194	0.001
Interbody fusion level	−0.101	0.084
Interbody fusion type	0.105	0.083
Pedicle screw fixation type	0.214	<0.001
Endplate breakage	0.037	0.542
ALL rupture	0.207	0.001

BMI = body mass index; Hb = hemoglobin; Hct = hematocrit, PT = prothrombin time; INR = international normalized ratio; aPTT = activated partial thromboplastin time; TBL = total blood loss; EBL = estimated blood loss; ALL = anterior longitudinal ligament.

**Table 5:** Multivariate linear regression analysis for hidden bleeding loss.

Coefficients for HBL	Unstandardized		Standardized	t	*p*
	Beta	SE	Beta		
Constant	−16.758	10.765		−1.557	0.121
TBL	0.996	0.006	0.899	174.895	<0.001
EBL	−0.935	0.011	−0.470	−81.788	<0.001
Pedicle screw fixation type	11.256	5.408	0.012	2.081	0.039

HBL = hidden bleeding loss; TBL = total blood loss; EBL = estimated blood loss.

## Data Availability

Data collected for this study, including individual patient data, will not be made available.
